# Intravenous Drug Use-Associated Infective Endocarditis in Pregnant Patients at a Hospital in West Virginia

**DOI:** 10.7759/cureus.17218

**Published:** 2021-08-16

**Authors:** Deena Dahshan, Mohamed Suliman, Ebad U Rahman, Zachary Curtis, Ellen Thompson

**Affiliations:** 1 Internal Medicine, Marshall University Joan C. Edwards School of Medicine, Huntington, USA; 2 Cardiology, Marshall University Joan C. Edwards School of Medicine, Huntington, USA; 3 Internal Medicine, St. Mary's Medical Center, Huntington, USA

**Keywords:** infective endocarditis, intravenous drug use (ivdu), high-risk pregnancy, west virginia, maternal and fetal outcome

## Abstract

Introduction

Due to high levels of intravenous drug use (IVDU) in West Virginia (WV), there are increasing numbers of hospitalizations for infective endocarditis (IE). More specifically, pregnant patients with IE are a uniquely challenging population, with complex management and a clinical course that further affects the health of the fetus, with high morbidity and mortality. Timely recognition and awareness of the most common bacterial causes will provide hospitals and clinicians with valuable information to manage future patients.

Methods

This retrospective study analyzed the clinical course of pregnant patients admitted with IE and IVDU history presenting at Cabell Huntington Hospital from 2013 to 2018. Inclusion criteria were women between 16 and 45 years of age confirmed to be pregnant by urine pregnancy test and ultrasonography with at least eight weeks gestation, with a first-time diagnosis of endocarditis and an identified history of IVDU. We excluded charts with pre-existing risk factors including a history of valvular disease, rheumatic heart disease, surgical valve repair or mechanical valve replacement, or a diagnosis of coagulopathies. The resulting charts were evaluated for isolated organisms, reported clinical course, and complications of the pregnancy.

Results

A total of 10 patients were identified, with methicillin-susceptible and methicillin-resistant *Staphylococcus aureus*, *Serratia marcescens*, *Haemophilus parainfluenza,* and *Enterococcus* *faecalis* species. Complications included loss of fetus (30%), septic embolization (40%), hemorrhagic stroke (10%), and transfer to outside facilities for cardiothoracic surgical intervention (40%).

Discussion

IE in pregnancy, while rare, has serious complications. In the context of the IVDU epidemic, it has an increasing impact on WV hospitals. A better understanding of the clinical course may allow for early diagnosis and guide the development of rational empiric therapies. More effective management of IE in pregnant patients can reduce complications and potentially improve maternal and fetal morbidity or mortality.

## Introduction

Infective endocarditis (IE) in pregnancy is a rare but life-threatening disease. Contributing intravenous drug use (IVDU) increases the risk of subacute IE. A study published in 2010 determined that one in five infants born in WV had significant drug exposure [1]. The incidence of maternal mortality in IE has previously been reported at 33% and fetal mortality at 29% among other complications [2]. Rising rates of IVDU in rural areas have shifted the burden onto rural hospitals’ resources and costs. A recent retrospective analysis of the economic impact of IVDU associated IE in Southern WV recognized over $13 million deficit hospital cost, with an increasing number of hospitalizations from 2008 to 2015 [3]. As patients do not always present with classic symptoms, timely recognition for appropriate management is key to minimize critical complications of IE in pregnancy, especially high-risk patients requiring urgent transfer for cardiothoracic surgery. 

IVDU-associated IE classically involves the tricuspid valve, and *Staphylococcus aureus* is the most common infectious agent recognized in these patients within the United States [3]. Other identified pathogens responsible for IE associated with IVDU include Streptococcus species and Gram-negative bacteria, *Pseudomonas,* and *Serratia marcescens*. In this study, we analyze the clinical course and bacterial causes of IE associated with IVDU in pregnant patients presenting at our hospital. Recognizing the associated complications and causal pathogens affecting pregnant patients hospitalized for IE with associated IVDU will provide hospitals and clinicians information to manage future patients accordingly.

## Materials and methods

After obtaining Institutional Review Board approval, we retrospectively identified pregnant patients who presented to Cabell Huntington Hospital (CHH) with IE from January 2013 through January 2018 using ICD 9 diagnosis codes combining “infective endocarditis,” “endocarditis” and “pregnancy.” Inclusion criteria were pregnant women confirmed with a pregnancy test and ultrasonography with at least eight weeks gestation, between 16 and 45 years of age, with the first-time diagnosis of endocarditis and identified history of injection drug use. We excluded charts with prior history of valvular disease, history of rheumatic heart disease, valve repair surgery or mechanical valve replacement, or diagnosis of coagulopathies including factor V Leiden thrombophilia, anti-thrombin 3 deficiency, antiphospholipid antibody syndrome, and Von Willebrand’s disease. The resulting patient charts were evaluated for isolated microorganisms, reported clinical course, and complications during admission. Data were collected by two members of the research team and screened and reviewed independently.

## Results

A retrospective chart review of patients presenting to CHH with IE identified 10 patients that met the criteria within hospital admissions for IE during pregnancy (Table 1).

**Table 1:** Clinical course summary. MSSA: methicillin-susceptible *Staphylococcus aureus*; MRSA: methicillin-resistant *Staphylococcus aureus*; y: years; d: days; HFrEF: heart failure with reduced ejection fraction; CTS: cardiothoracic surgery.

Age	Organism	Pathology	Treatment	Length of stay	Follow up	Complications
24y	MSSA	Tricuspid valve	Nafcillin	18d	No record of follow up	None
29y	MSSA	Tricuspid valve	Vancomycin	44d	No maternal complications on follow up	Abscesses in lung and spine Placenta abruptio Non-viable fetus at 22.5 weeks
20y	MRSA	Tricuspid valve	Vancomycin	18d	Patient did not present for follow up	Tricuspid valve septal leaflet perforation Fetal demise at 11 weeks
27y	MSSA	Right ventricle vegetation	Nafcillin; 6 weeks cefazolin	6d	Transferred to outside facility for CTS	Emergent cesarean section with twins at 29.3 weeks
24y	Serratia marcescens	Tricuspid valve Hepatic vein systolic flow reversal	Meropenem	24d	Lost to follow up	Gestational hypertension with HFrEF 45% Cesarean section at 37 weeks
33y	Haemophilus parainfluenza	Tricuspid valve Dilated pulmonary artery	Vancomycin Rocephin	1d	Transferred to outside facility for CTS	Septic emboli High grade cervical dysplasia status post cone biopsy Nonviable fetus at 22 weeks
31y	Enterococcus faecalis	Mitral valve	Zosyn	2d	Transferred for CTS	Septic emboli Intracranial hemorrhage resulting contralateral plegia
24y	Serratia marcescens	Tricuspid valve	Ertapenem	24d	Lost to follow up	Presented at 20 weeks gestation, unaware of pregnancy
28y	MSSA	Pulmonic valve vegetation	Cefazolin; oral Linezolid	27d	Lost to follow up	Cholestasis of pregnancy Pre-Eclampsia Delivered 36 weeks
25y	MRSA	Tricuspid valve	Vancomycin	2d	Transferred to outside facility for CTS	Septic emboli Breech vaginal delivery at 40 weeks

**Figure 1:**
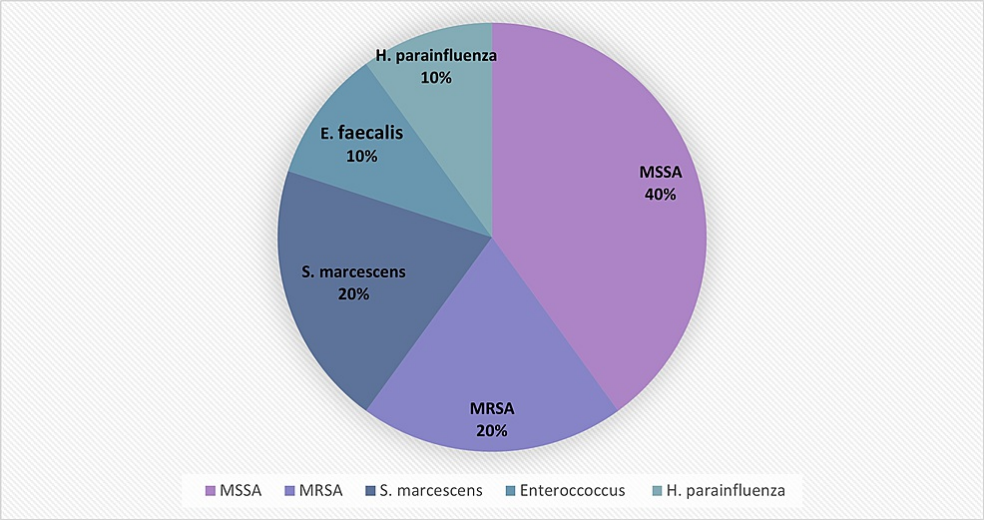
Summary of pathogen etiology. MSSA: methicillin-susceptible *Staphylococcus aureus*; MRSA: methicillin-resistant *Staphylococcus aureus*.

Isolated infective organisms included methicillin-susceptible and methicillin-resistant *Staphylococcus aureus*, *Serratia marcescens*, *Haemophilus parainfluenza* as well as *Enterococcus faecalis* species (Figure 1).

Patient ages ranged from 20 to 33 years. Length of hospital stay for patients that did not require transfer to an outside facility for cardiothoracic surgery ranged from 18 to 44 days. Resulting clinical course following admission found 40% of patients required immediate cardiothoracic surgery (CTS) evaluation and transfer to an outside facility (Table 2).

**Table 2:** Summary of complications.

Clinical course during admission	Percent of patients
Immediate cardio-thoracic surgery evaluation	40%
Septic embolism	40%
Hemorrhagic stroke with contralateral plegia	10%
Fetal mortality	30%

Septic emboli occurred in 40% of patients with endocarditis, with one patient’s case further complicated by an intracranial hemorrhage. A total of 30% of pregnancies resulted in an unviable fetus, with gestation periods ranging from 11 to 22.5 weeks.

## Discussion

Our chart review demonstrates significant maternal and fetal complications for pregnant patients with IVDU related IE presenting at CHH. Long-term hospitalization of high-risk pregnancy patients allows for critical maternal and fetal surveillance and intervention. Receiving appropriate health care before and during pregnancy decreases the risk for complications. However, it is not uncommon that patients using opioids have menstrual irregularities related to opioid use and unplanned pregnancies. Individuals with substance use are often affected by multiple risk factors and a lack of support that may further complicate the pregnancy. Drug use-associated IE in the South Eastern United States comprised 14% of hospitalizations in 2009 and increased to 56% of hospitalizations in 2014 [4]. A five-year study at the West Virginia University Medical Center from 1960 to 1965 reported 25 cases of endocarditis of which 18 patients had underlying rheumatic heart disease with no cases mentioning substance use related IE [5]. Our retrospective review at CHH exclusively identifying IVDU associated IE cases in pregnant patients admitted from 2014 to 2018 found 10 cases that demonstrate a significant rise in drug use associated IE when compared to prior incidences of endocarditis in the general population.

Patients with IVDU with IE have tricuspid valve involvement in over 70% of cases without underlying pre-existing cardiac disease in previously reported literature [6]. Similarly, we found that 70% of our cases involved the tricuspid valve, but other cases included the mitral valve and pulmonic valve. We excluded cases involving rheumatic heart disease and prior valve disease in our retrospective review as these are recognized inherent risk factors for endocarditis and to further focus on this rising vulnerable population affected by substance use. Opiate injection use in rural communities appears to be a major contributor [4]. Patients with IE related to substance use have longer hospital stays with almost twice the cost of admission compared to patients with IE unrelated to substance use [7]. We did not compare pregnant patients with IE unrelated to IVDU, but we believe IE associated with IVDU similarly complicates clinical course in pregnant patients. In our review, we identified 40% of cases involved methicillin-susceptible *Staphylococcus aureus *and 20% incidence of methicillin-resistant *Staphylococcus aureus *among pregnant patients with associated IVDU admitted for IE. This corresponds to the most common etiology of IE with IVDU [3]. 

Pregnant patients with IE have serious complications from resulting embolic events and mycotic aneurysms [8]. In our study, 40% of patients had embolic events, with one case presenting with an extensive hemorrhagic stroke with contralateral plegia (Table 2). Patients may present with a low-grade fever, shortness of breath, heart murmur, petechiae, anemia, and embolic events with associated valve vegetations [8]. Prompt recognition and management of IE in pregnant patients are critical to minimize serious sequelae [9]. Serious sequelae of IE embolic events include splenic infarction and pulmonary embolism, both life-threatening [9]. Mycotic aneurysms can occur in any major vessel, with reported cases of rapid progression and rupture of mycotic cerebrovascular aneurysms leading to lethal intra-parenchymal or subarachnoid hemorrhages [10]. In the patient with intracranial hemorrhage, neurology recommended delaying labor induction for malignant cerebral edema following the stroke, while obstetrics and gynecology recommended delivery for a viable fetus. This patient was transferred to an outside facility due to lack of cardiothoracic surgery (Table 1). While there were not any maternal death during the hospitalization admission for the patients with complications of septic emboli recorded in our review, we did not access records for these high-risk patients transferred to outside facility for cardiothoracic surgery to follow maternal or fetal mortality after discharge. 

The incidence of IVDU associated IE continues to rise, with increased readmission for endocarditis, septicemia, and substance use presenting a challenge to existing management of IE. We did not evaluate readmission rates in this review. As not all patients present with the classical manifestations of IE bacteremia, fever, cardiac murmur, and peripheral vascular findings and so it can often be difficult to promptly diagnose IE if clinicians carry a low index of suspicion [11]. Complications and interventions of IE during pregnancy affect both the mother and the fetus, with morbidity and mortality rates increasing for the fetus [12]. Our review found 30% fetal mortality during hospital admission and severe maternal complications (Table 2). 

Reported indications for immediate transfer for cardiothoracic surgery evaluation in pregnant patients with IE include clinical deterioration, evidence of vegetation enlargement, infection uncontrolled by medical intervention, and increasing valve dysfunction [8]. While surgery is required in 25%-50% of acute and 20-40% of convalescent infections in general population of patients with IE, it is an even higher risk with challenging management in patients with intravenous drug use (IVDU) and poor compliance with treatment [6]. Urgent cardiac surgery is required in two-thirds of IE cases in pregnant patients due to valve deterioration, with higher association of fetal deaths affecting patients undergoing cardiac surgery in early gestational age [8]. Rural hospitals may not have adequate resources for managing patients needing surgery, and these patients must be promptly transferred to outside centers for urgent cardiothoracic surgical intervention. In this study, 40% of patients required transfer to an outside facility for urgent cardiothoracic surgery (Table 2). Previous cases of open-heart surgery indicated in IE complicating pregnancy have been safely performed but require extensive monitoring and protection strategies implementation for the health and safety of the mother and the fetus [13]. We did not follow surgical mortality at outside facilities. 

A limitation in this study is the number of patients lost to follow-up, with an incomplete report of complications and resulting clinical course in our records. Five of the 10 patients in our study were lost to follow-up after discharge, and four required transfer of care to an outside facility for cardiothoracic surgery. Continuity of care would have allowed for a more comprehensive understanding of each patient’s IE and pregnancy course. This may be an inherent limitation specific to the patient population we analyzed, lacking support and resources compared to the general population as well as our facility lacking cardiothoracic surgery. Another limitation was focusing solely on the maternal clinical course and pregnancy complications. We recognize that our study could have expanded to assess for prolonged neonatal intensive care hospitalization and other infant complications following delivery consequent to maternal IE with IVDU. However, our primary focus was the maternal clinical course during admission for IVDU associated IE with pregnancy and we did not follow complications of the infant after delivery. Another weakness in this study was not including a comparison of IE cases in patients without IVDU or pregnancy admitted to our hospital. We recognize IE during pregnancy is rare, and with the increasing IVDU in WV, this presentation carries a high maternal and fetal morbidity and mortality. We highlight this presentation in comparison to prior published literature of IE incidence in WV with no previous mention of substance use to demonstrate the increasing need for intervention and provide hospitals information to manage future patients expectingly.

Currently, harm reduction programs in WV provide education and services to reduce IVDU complications and the potential for needle sharing. The prevalence of drug use in pregnant WV patients is 2- to 3-fold higher than estimates from voluntary reporting with 20% of infants identified to have a significant drug exposure not captured by conventional reporting instruments [1]. Patients with IVDU are at increased risk for IE, they have a higher incidence of recurrence, and a higher mortality than patients with IE without associated IVDU [14]. Access to resources can be even more difficult for pregnant women with substance use disorder, especially when using opioids. Buprenorphine and methadone rehabilitation clinics are options to improve maternal safety compared to uncontrolled opioid use in pregnancy [15]. Harm reduction programs including needle exchange programs can further reduce infection incidence among severely addicted injection drug users [16]. Interventions to reduce IE related to IVDU in pregnant patients can decrease maternal and fetal morbidity and mortality, reduce extensive hospitalization, and lessen the significant financial costs.

## Conclusions

With the rising IVDU epidemic in WV as well as the rising incidence of IE, especially in pregnancy, further preparation and intervention are warranted to appropriately identify patients before they present with acute IE in the hospital. Isolated infective organisms included methicillin-susceptible and methicillin-resistant *Staphylococcus aureus, Serratia marcescens, Haemophilus parainfluenza* as well as* Enterococcus faecalis* species. Complications in patients with IE in pregnancy have severe consequences. A better understanding of epidemiology and associated risk factors is needed to manage future patients more efficiently. Strategies to prevent IVDU associated IE in pregnancy, optimize treatment, and reduce morbidity are necessary to improve maternal and fetal outcomes.
